# Spontaneous Supraceliac Isolated Abdominal Aortic Dissection Sparing Major Visceral and Renal Vessels and Presenting as Chronic Limb Ischemia

**DOI:** 10.1155/2011/890204

**Published:** 2011-04-28

**Authors:** Sean O. Z. Bello, Ilias Kouerinis, Woolagasen Pillay

**Affiliations:** Department of Vascular Surgery, Doncaster Royal Infirmary, Armthorpe Road, Doncaster DN2 5LT, UK

## Abstract

Aortic dissections that originate from isolated tears in the abdominal aorta are uncommon. Rarer still are cases of isolated abdominal aortic dissections arising in suprarenal locations, as most appear from infrarenal intimal defects. We present a quite unusual case of a spontaneous supraceliac isolated abdominal aortic dissection sparing the renal and mesenteric arteries and manifesting as chronic rather than acute limb ischemia. The atypical presentation of this case led to repeated misdiagnosis with consequent loss of part of the patient's limb. Better illustration of the natural history of this ill-defined pathology is needed to aid understanding and improve patient care.

## 1. Introduction

Spontaneous isolated abdominal aortic dissection (IAAD) (not associated with trauma or with descending thoracic aortic dissection) is rare and accounts for less than 2% of all aortic dissections [1]. Spontaneous suprarenal IAADs are even more rarely encountered as most appear from infrarenal intimal defects. This subgroup of IAAD is often associated with poorer prognosis as the dissection may interfere with blood flow through the renal and mesenteric arteries predisposing to ischemia of their respective organs and consequent high mortality [2, 3]. We present a quite unusual case of spontaneous supraceliac IAAD sparing both renal and mesenteric vessels, and manifesting atypically as chronic rather than the more typical acute limb ischemia in a patient with chronic paraplegia secondary to previous spinal surgery for chondrosarcoma.

## 2. Case Report

A 41-year-old paraplegic presented to the emergency department with a ten-day history of worsening pain in his left forefoot. His symptoms began after he injured his left hallux whilst swimming, and he had just completed three days of antibiotics for apparent cellulitis over the toe with no improvement in his symptoms.

He was a nonsmoker and had no history of intermittent claudication, DVT/PE, calf tenderness, arrhythmia, diabetes, hypertension, or high cholesterol. He had spinal surgery 2 years earlier for chondrosarcoma, and this rendered him paraplegic.

He was normotensive, afebrile, and in sinus rhythm. Clinical examination revealed a dusky discoloration to his left hallux with a cold and tender forefoot. His right foot was unremarkable.

**Figure 1:**
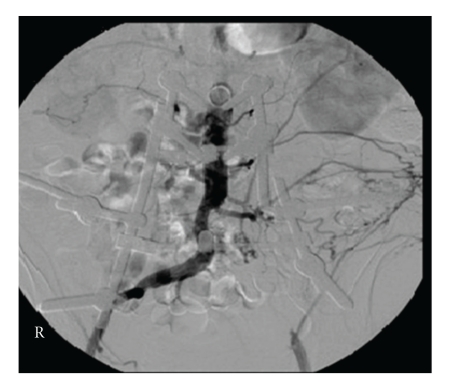
Right transfemoral angiogram at presentation showing occlusion to left common iliac artery extending into the left external iliac artery.

He had normal pulses with good doppler signals in the right leg but no palpable pulses from the left common femoral artery down the left leg. Doppler signals were also absent on the left. His abdomen was soft and nontender, and he had no other detectable clinical signs. A provisional diagnosis of left iliofemoral artery thrombosis with possible distal embolic disease was made. He proceeded to have an urgent right transfemoral angiogram which revealed normal-looking right lower limb vessels with no atherosclerotic lesions or changes. There was, however, a cutoff of contrast at the origin of the left common iliac artery (CIA) extending into the left external iliac artery (EIA). There was reconstitution of the left common femoral artery (CFA), profunda femoris artery (PFA), and superficial femoral artery (SFA). The above knee popliteal artery (PA) was occluded with reconstitution of a single vessel runoff at mid leg, most probably the posterior tibial artery (Figure 1).

The impression at this point was that of a thrombotic event at the left CIA with showering of emboli to the distal SFA and PA. The patient proceeded to theatre the next day for a left iliac/femoral artery embolectomy +/− stent insertion +/− thrombolysis.

A fogarty catheter passed through an arteriotomy of the left CFA was used to retrieve large amounts of thrombi, but there was little improvement to blood flow. Further embolectomy was carried out until no more thrombus was retrievable, but flow through the left CFA remained poor. A strong right common femoral pulse, however, remained and as such an impression of probable undiagnosed aortic dissection was made.

The left CFA was ligated, and a right-to-left femorofemoral crossover using an 8mm polytetrafluorethylene (PTFE) graft was established with good results.

**Figure 2:**
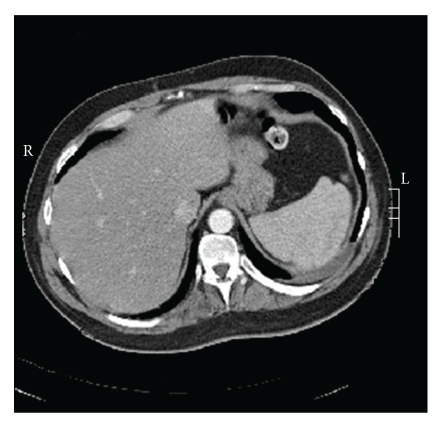
Post-op contrast CT abdomen showing normal abdominal aorta at its origin.

**Figure 3:**
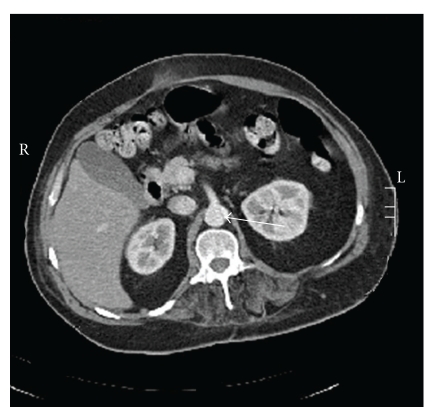
Post-op contrast CT abdomen showing supraceliac aortic dissection (white arrow).

Abdominal computed tomography (CT) after surgery confirmed the presence of a left anterolateral abdominal aortic dissection from just above the celiac level of the celiac axis extending down into a ligated left common iliac artery. The dissection lies to the left of the celiac axis curving anteriorly to the left and sparing the left and right renal arteries which appeared patent behind the dissection (Figures 2 and 3). A previous abdominal CT scan taken 1 year after his spinal surgery showed a normal aorta.

This patient subsequently required amputation of his left forefoot but has otherwise made good progress and is now having regular outpatient surveillance of his aortic dissection and close blood pressure monitoring.

## 3. Discussion

Spontaneous isolated abdominal aortic dissection is very rare. In the International Registry of Acute Aortic Dissection (IRAD) (the largest group of patients treated for acute aortic dissections), only about 1.3% of the enrolled patients were identified as having isolated dissections of the abdominal aorta [4]. Furthermore, a recent meta-analysis of all English language articles regarding abdominal aortic dissection found only 73 reported cases of spontaneous IAAD [5]. In these cases, the dissection flap predominantly originated below or at the level of the renal arteries; very few had the intimal tear in the suprarenal aorta as illustrated in this case. 

The mean age at presentation of IAAD is in the fifth or sixth decade of life. It is more common in Caucasians, and men are affected twice as commonly as women [4]. Identified risk factors include hypertension, smoking, dyslipidemia, connective tissue disease, and trauma [6]. The natural history of IAAD is yet to be clearly defined, and in many cases, the presenting clinical symptoms are nonspecific [7]. It, however, most commonly presents with sudden onset of abdominal pain radiating to the back and to the buttock but could also present with acute lower limb ischemia (or chronic lower limb ischemia as this case describes), or symptoms of visceral malperfusion and renal ischemia. These last two presentations are manifestations of suprarenal involvement and carry much poorer prognosis [8]. The mortality rate of patients with renal ischemia is reported to be 50 to 70%, whilst mortality figures in mesenteric ischemia can be as high as 87% [3]. 

Paraplegia after spontaneous dissection of the abdominal aorta has also been reported [9]. This may be a result of obstruction to blood flow through the Adamkiewicz artery (the largest anterior segmental medullary artery) that supplies the lower two-thirds of the spinal cord. Our patient's paraplegia was, however, not associated with his aortic disease. He had been paraplegic for two years before this presentation. His paraplegia was secondary to spinal surgery to remove a large chondrosarcoma that had invaded the 1st and 2nd sacral segments via the spinal canal and extended superiorly within the spinal canal to the level of the midbody of L5. Although he admittedly had extensive spinal surgery with the institution of an internal fixation device to his lumbar spine making it difficult to completely exclude this as a contributory factor to his dissection, abdominal CT scan carried out one year after his spinal surgery revealed a normal abdominal aorta. 

Accurate early diagnosis is paramount in spontaneous IAAD, and contrast CT scan is the investigation of choice. Whilst our case did present with a recognised clinical feature of this pathology albeit atypical, that is, chronic rather than acute lower limb ischemia, he had no abdominal symptoms or signs, and the nature of the onset of his symptoms coupled with his lack of discernible risk factors made the diagnosis difficult. There was no indication for a CT scan at presentation.

Management of patients with IAAD is either medical or operative. Indications for operative intervention include aortic rupture, unremitting pain, associated aortic aneurysm, prevention of future aneurysmal change, and visceral, renal, or lower extremity ischemia. Asymptomatic patients with a nondilated aorta are treated with antihypertensive medication [10, 11]. Operative management is via open (graft) or endovascular (stent) repair of the affected abdominal aorta. This decision is greatly influenced by anatomical conditions, the patient's comorbidities, and the surgeon's experience. Where the dissection extends to the iliac arteries, aorto-bifemoral/monofemoral grafting is the operation of choice. 

Repair of supraceliac IAAD is more challenging. Elliott et al. described the first successful repair of a spontaneous suprarenal abdominal aortic dissection by graft insertion with obliteration of the entry tear. Before this, operative intervention of spontaneous suprarenal abdominal aortic dissection met with generally poor results [10]. More recently, less invasive endovascular approaches have been proposed. These techniques are balloon fenestration and intraluminal stent procedures. Percutaneous balloon aortic fenestration involves obtaining arterial access at the common femoral artery, and under intravascular ultrasound (IVUS) or arteriographic guidance, fenestration is accomplished by puncturing the intimal flap with an intravascular needle followed by balloon dilatation (at least a 15 mm balloon) of the flap [12]. This provides local blood flow between the true and false lumens by creating a tear in the intervening dissection septum thus achieving an equalization of pressures between the two lumens. The degree of obstruction of the branch vessel ostia is also examined, and stents may be added to optimize flow. A stent graft may or may not be required within the aorta. The technical success of this procedure is estimated to be about 90%, with visceral vessel involvement, more so than the renal vessels, predicting worse outcome [12]. 

Described complications of percutaneous fenestration include aneurysm formation, transmural perforation during the creation of the fenestration, and manipulation of the intimal flap, which can propagate the dissection or cause occlusion of previously patent vessels. Furthermore, laboratory studies have shown that balloon fenestration tears are typically along a transverse orientation relative to the longitudinal axis of the aorta [13]. As such, in some cases, the transverse tear could circumferentially transect an aortic septum (when a septal tube is present) and result in intimo-intimal intussusceptions with resultant occlusion of distal vessels [14]. 

Balloon fenestration and intraluminal stent procedures remain promising, but only small series have so far documented their use and long-term data are not yet available. Optimal patient selection for these procedures has also not yet been described [15]. Suspicion of our patient's diagnosis was only arrived at on the operating table during which femorofemoral crossover revascularisation of the left system had to be established due to lack of adequate analysis of the position or extent of the dissection flap. Fortunately, his dissection did not require further operative intervention.

## 4. Conclusion

Spontaneous supraceliac IAAD is a very rare but potentially life- or limb-threatening condition particularly if misdiagnosed. It may have a number of clinical presentations with potentially serious adverse effects and should thus be considered in the differential diagnosis of any patient with an acute onset of abdominal pain radiating to the back and the buttocks together with presence or absence of a pulsatile abdominal mass, signs of limb ischemia, or discernible risk factors. The unusual presentation of this case led to difficulty in early diagnosis culminating in loss of part of the patients' limb. Better illustration of the natural history of this ill-defined pathology is needed to aid understanding and improve patient care.
